# Astaxanthin Inhibits p70 S6 Kinase 1 Activity to Sensitize Insulin Signaling

**DOI:** 10.3390/md18100495

**Published:** 2020-09-28

**Authors:** Chunmei Li, Bixia Ma, Junhong Chen, Yoonhwa Jeong, Xiulong Xu

**Affiliations:** 1 College of Veterinary Medicine, Yangzhou University, Yangzhou 225009, China; licm@yzu.edu.cn; 2 College of Food Science and Engineering, Yangzhou University, Yangzhou 225009, China; mabixia@vazyme.com; 3 Institute of Animal Science and Technology, Jinling Institute of Technology, Nanjing 211169, China; chenjunhong@jit.edu.cn; 4 Research Center for Industrialization of Natural Nutraceuticals, Dankook University, Cheonan 31116, Korea; yjeong@dankook.ac.kr; 5 Department of Food Science and Nutrition, Dankook University, Cheonan 31116, Korea; 6 Institute of Comparative Medicine, Yangzhou University, Yangzhou 225009, China; 7 Jiangsu Co-innovation Center for Prevention and Control of Important Animal Infectious Diseases and Zoonosis, Yangzhou University, Yangzhou 225009, China; 8 Institutes of Agricultural Science and Technology Development, Yangzhou University, Joint International Research Laboratory of Agriculture and Agri-Product Safety, The Ministry of Education of China Yangzhou University, Yangzhou 225009, China

**Keywords:** astaxanthin, S6K1, GLUT4, insulin receptor, AKT, PI-3 kinase, glucose uptake

## Abstract

Astaxanthin (AST) is a carotenoid with therapeutic values on hyperglycemia and diabetic complications. The mechanisms of action of AST remain incompletely understood. p70 S6 kinase 1 (S6K1) is a serine/threonine kinase that phosphorylates insulin receptor substrate 1 (IRS-1)^S1101^ and desensitizes the insulin receptor (IR). Our present study aims to determine if AST improves glucose metabolisms by targeting S6K1. Western blot analysis revealed that AST inhibited the phosphorylation of two S6K1 substrates, S6^S235/236^ and IRS-1^S1101^, but enhanced the phosphorylation of AKT^T308^, AKT^S473^, and S6K1^T389^ by feedback activation of the phosphatidylinositol-3 (PI-3) kinase in 3T3-L1 adipocytes and L6 myotubes. In vitro kinase assays revealed that AST inhibited S6K1 activity with an IC_50_ value of approximately 13.8 μM. AST increased insulin-induced IR tyrosine phosphorylation and IRS-1 binding to the p85 subunit of PI-3 kinase. Confocal microscopy revealed that AST increased the translocation of the glucose transporter 4 (GLUT4) to the plasma membrane in L6 cells. Glucose uptake assays using a fluorescent dye, 2-NBDG (2-*N*-(Nitrobenz-2-oxa-1,3-diazol-4-yl)amino)-2-deoxyglucose), revealed that AST increased glucose uptake in 3T3-L1 adipocytes and L6 myotubes under insulin resistance conditions. Our study identifies S6K1 as a previously unrecognized molecular target of AST and provides novel insights into the mechanisms of action of AST on IR sensitization.

## 1. Introduction

Diabetes mellitus is a highly prevalent chronic disease characterized by high blood glucose levels and many devastating complications [1,2]. It is estimated to contribute to 11.3% of deaths globally in 2019 [3,4]. According to the International Diabetes Federation Diabetes Atlas, the number of global diabetes cases may reach 578 million in 10 years. Among them, type 2 diabetes mellitus (T2DM) accounts for 90% of cases [5]. Dietary interventions, medications, and surgery have been used to control T2DM [6]. However, because of the reduced efficacy or complications of long-term medications, hyperglycemia is poorly controlled in many patients [7,8]. It is imperative to develop novel strategies to prevent and treat diabetes. 

Extensive studies in the past few decades have greatly advanced the understanding of the pathogenesis and the integrative biology of T2DM [4,9]. Insulin binding to the IR triggers IR autophosphorylation and insulin receptor substrate (IRS) tyrosine phosphorylation [9,10]. Tyrosine-phosphorylated IRS transduces a cascade of downstream signals through the p85 subunit of the PI-3 kinase, leading to serine phosphorylation of the protein kinase B (AKT) and the mammalian target of rapamycin (mTOR) [9,10]. AKT facilitates glucose uptake by increasing GLUT4 translocation to the plasma membrane (PM) [10,11]. S6K1, a serine/threonine protein kinase downstream of mTOR in the insulin signaling pathway, plays a critical role in driving insulin resistance [9,12]. S6K1-deficient mice fed a high-fat diet do not develop obesity and diabetes [13,14]. Under overnutrient conditions, S6K1 attenuates the activation of the PI-3 kinase pathway by phosphorylating IRS-1 and suppressing its function to negatively regulate insulin signaling [12,13,14]. 

The consequence of persistent overnutrition is insulin resistance, in which the metabolic tissues such as the liver, muscle, and fat do not respond or poorly respond to insulin stimulation to lower blood glucose levels due to IR desensitization [9,10]. Insulin resistance can be recapitulated in vitro in cell culture. For example, when 3T3-L1 adipocytes and L6 myotubes are cultured in media containing high concentrations of amino acids, mTOR is highly and constitutively activated. mTOR phosphorylates and activates S6K1, which then phosphorylates IRS-1^S1101^, leading to poor activation of its downstream PI-3 kinase as evidenced by weak binding of the PI-3 kinase to IRS-1 and weak AKT phosphorylation upon insulin stimulation [9,10]. Several natural products such as gingerenone A and evodiamine, which inhibit S6K1 activity, can increase glucose uptake or improve glucose tolerance [15,16]. 

Astaxanthin (AST) is a natural, pigmented xanthophyll carotenoid commonly present in microalgae, yeasts, salmon, trout, krill, and other seafood [17,18]. *Haematococcus pluvialis* (*H. pluvialis*), a freshwater unicellular alga, contains the highest levels of natural AST [18,19]. The therapeutic and nutritional values of AST have attracted the widespread attention of the scientific community and the public in the past two decades [17,20]. AST is best known for its anti-oxidative activity [17]. Its ability to scavenge reactive oxygen species (ROS) in humans is higher than vitamin E and β-carotene [21,22]. AST possesses several other biological effects such as immunity-boosting, anti-inflammatory, anti-cancer, and anti-aging activities [18,23,24,25]. Numerous studies have shown that AST possesses potential anti-hyperglycemic effects in mouse models and humans [20]. For example, AST enhances insulin signaling, improves glucose metabolism, and prevents steatohepatitis in the liver of insulin-resistant mice [22,26,27,28] and individuals with prediabetes conditions [29]. AST induces AKT phosphorylation and enhances GLUT4 translocation to the plasma membrane and glucose uptake in L6 myotubes [26,30]. However, the mechanisms by which AST activates AKT and its molecular targets remain largely unknown [30,31]. Our present study provides evidence that AST functions as an S6K1 inhibitor to induce feedback activation of the PI-3 kinase pathway, subsequently activating AKT to increase GLUT4 translocation to the cell membrane and enhance glucose uptake in 3T3-L1 adipocytes and L6 myotubes. Our study identifies a new molecular target of AST and provides novel insights into how AST sensitizes the IR and improves glucose metabolism. 

## 2. Results

### 2.1. AST Induces Feedback Activation of the PI-3 Kinase Pathway

**Figure 1:**
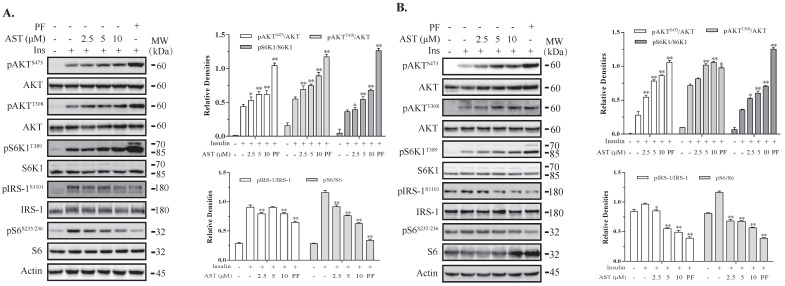
Astaxanthin (AST) induces feedback activation of the PI-3 kinase pathway. 3T3-L1 adipocytes (**A**) and L6 myotubes (**B**) were starved of serum overnight and for 2 h, respectively. After preincubation in the absence or presence of the indicated concentrations of AST or PF-4708671 (10 μM) for 4 h, the cells were left unstimulated or stimulated with (100 nM) for 10 min. Cell lysates were prepared and analyzed for the phosphorylation of IRS^S1101^, AKT^T308^, AKT^S473^, S6K1^T389^, and S6^S235/236^ with their specific antibodies and re-probed with antibodies against total proteins. The density of the phosphorylated protein bands was analyzed by using NIH Image-J software and normalized by the arbitrary units of total proteins. Data are expressed as the mean ± standard derivation (SD) of three experiments. * *p* < 0.05, ** *p* < 0.01, compared to the insulin-stimulated controls.

The PI-3 kinase pathway is activated by the IR and plays an important role in glucose metabolism [9]. We first evaluated the effect of AST on the PI-3 kinase pathway in 3T3-L1 adipocytes and L6 myotubes, two cell types that have been widely used to study insulin resistance. As shown in Figure 1A,B, insulin induced phosphorylation of IRS^S1101^, AKT^T308^, AKT^S473^, S6K1^T389^, and S6^S235/236^ in 3T3-L1 adipocytes. AST enhanced the phosphorylation of AKT^T308^, AKT^S473^, and S6K1^T389^ in a dose-dependent manner, but suppressed insulin-induced S6^S235/236^ in 3T3-L1 adipocytes in a dose-dependent manner (Figure 1A,B). AST inhibited IRS^S1101^ phosphorylation more effectively in L6 myotubes than in 3T3-L1 adipocytes. PF-4708671, an S6K1-specific inhibitor included as a positive control, increased AKT^T308^, AKT^S473^, and S6K1^T389^ phosphorylation but significantly decreased IRS^S1101^ and S6^S235/236^ phosphorylation in both cell types (Figure 1A,B).

### 2.2. AST Inhibits S6K1 Activity 

**Figure 2:**
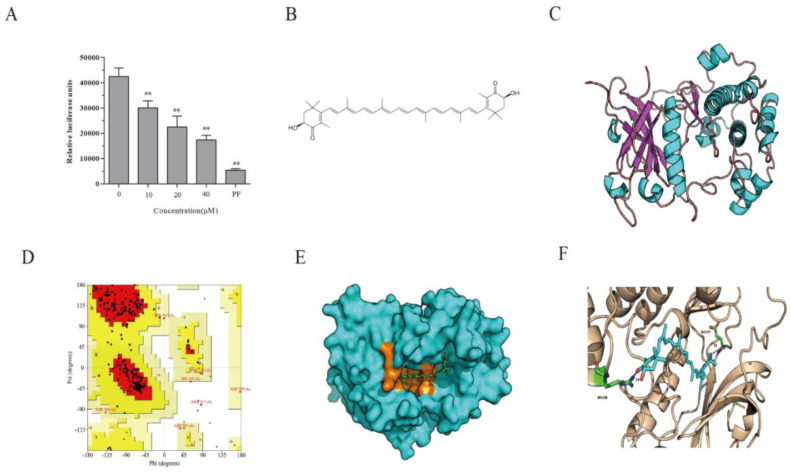
AST inhibits S6K1 activity. (**A**) The ability of AST to inhibit S6K1 activity was assayed by an ADP-Glo^TM^ kinase kit. (**B**) Chemical structure of AST; in vitro S6K1 kinase assay. (**C**) The predictive model of S6K1 (red: β-folds; green: α-helices). The model was built using a Swiss-Model Server. (**D**) Ramachandran plot of the S6K1 3D model. The Ramachandran plot was performed with a PROCHECK program. (**E**) AST binds to S6K1 in the ATP binding pocket (orange). (**F**) Interactions between AST and S6K1. AST binds to S6K1 by two hydrogen bonds, and the amino acid residues involved in the formation of hydrogen bonds were Glu173 and Arg298. The molecular docking diagram was performed by using MGLTools 1.5.6., PyMOL 1.2, and AutoDock Vina 1.1.2. ** *p* < 0.01, compared to the untreated control.

The ability of AST to decrease S6 and IRS-1 phosphorylation but increase AKT and S6K1 phosphorylation suggests that S6K1 is the molecular target of AST. We conducted an in vitro kinase assay and found that AST indeed inhibited the activity of recombinant S6K1 in a dose-dependent manner, with an IC_50_ value of 18.3 μM (Figure 2A). PF-4708671 at the concentration of 10 μM inhibited S6K1 activity by 87.2%.

### 2.3. S6K1 Structure Prediction and Model Validation 

The chemical structure of AST is shown in Figure 2B. The amino acid sequence of the catalytic domain of S6K1 was retrieved from the Uniprot database. By aligning target sequences with template structures (PDB 4L44), the template coverage and sequence consistency of the template were 65% and 99.71% identical, respectively. Modeling with a Swiss-Model Server reveals a 3D structure of S6K with 13 helices and 7 folds (Figure 2C). The stereochemical quality and accuracy of the predicted model were then evaluated using a Ramachandran Map with PROCHECK (Version 4, University of California at Los Angeles, Los Angeles, CA, USA) analysis. In the Ramachandran plot (Figure 2D), the red and yellow regions in the graph represent the most allowed regions, which reach 90% of the total protein residues (excluding glycine, which is represented by triangles). This is indicative of the accuracy of the protein structure model with a high-quality. With the phi and psi angles, 88.7% (258 amino acids) of the amino acids excluding glycine and proline resided in the most favored regions, 8.9% (26 amino acids) were in additional allowed regions, 1.7% (5 amino acids) were in the generally allowed regions, and 0.7% (2 amino acids) residues were in the disallowed regions. The proportion of the homologous modeling structure of S6K1 reached 97.6% in this case. The primary stereochemical properties of the homologous modeling structure were determined to be of high fidelity.

### 2.4. Docking Fitting 

Based on the model of the S6K1 structure, we employed software to determine if AST can “dock” into S6K1. The crystal structure is illustrated in Figure 2E. AST fits as an S6K1 inhibitor to dock into the ATP-binding pocket (orange) of the kinase domain. AST was bound to S6K1 by its two hydrogen bonds with the hydrogen bonds of Glu173 and Arg298 (Figure 2F). The binding capacity of AST to S6K1 is −8.5 kcal/mol. 

### 2.5. AST Sensitizes The IR by Feedback Activating The PI-3 Kinase Pathway

**Figure 3:**
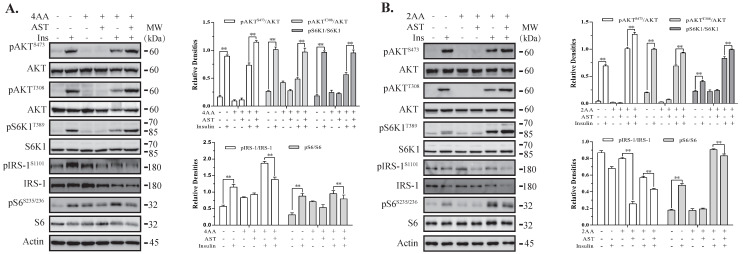
AST induces feedback activation of the PI-3 kinase pathway under insulin resistance conditions. 3T3-L1 adipocytes (**A**) and L6 myotubes (**B**) were starved of serum overnight and for 2 h, respectively. These cells were incubated for 4 h in an essential balanced salt solution (EBSS) in the absence or presence of AST (10 μM), minus or plus high concentrations of amino acids (AA). The cells were then left unstimulated or stimulated with insulin (100 nM) for 10 min. Cell lysates were prepared and analyzed for the phosphorylation of IRS^S1101^, AKT^T308^, AKT^S473^, S6K1^T389^, and S6^S235/236^ with their specific antibodies and then re-probed with antibodies against their total proteins. The density of the phosphorylated protein bands was analyzed by using NIH Image-J software and normalized by the arbitrary units of total proteins. Data represent the mean ± SD of three experiments. ** *p* < 0.01, compared to insulin-stimulated controls.

Having verified AST as an S6K1 inhibitor, we further investigated the effect of AST on insulin signaling in the cells cultured under insulin resistance conditions. 3T3-L1 adipocytes and L6 myotubes were incubated for 4 h in the absence or presence of AST (10 μM), minus or plus 4-fold (4×) and 2× concentrated amino acids (AA), respectively. Of note, 4× and 2× AA were diluted from a 100-fold-concentrated AA stock. Insulin readily induced AKT^T308^, AKT^S473^, S6K1^T389^, and S6^S235/236^ phosphorylation in both 3T3-L1 adipocytes and L6 myotubes in the absence of amino acids (Figure 3A,B). Insulin increased AKT^T308^, AKT^S473^, and S6K1^T389^ phosphorylation in 3T3-L1 adipocytes in the presence of high AA at a significantly lower magnitude than its absence (Figure 3A). AST significantly increased the levels of AKT^T308^, AKT^S473^, and S6K1^T389^ phosphorylation in the insulin-stimulated adipocytes cultured in the presence of 4× AA. Similar observations were made with L6 myotubes (Figure 3B).

### 2.6. AST Increases IR Tyrosine Phosphorylation and IRS-1 Binding to the p85 Subunit of PI-3 Kinase

**Figure 4:**
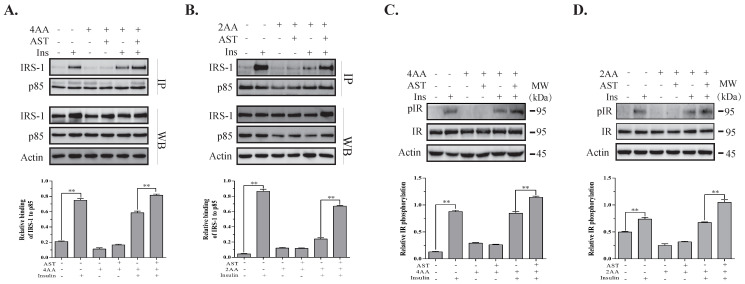
AST sensitizes the insulin receptor (IR). 3T3-L1 adipocytes (**A**,**C**) and L6 myotubes (**B**,**D**) were starved of serum overnight and 2 h, respectively. 3T3-L1 adipocytes and L6 myotubes were then incubated for 4 h in EBSS or EBSS containing 4× and 2× AA, respectively, and in the absence or presence of 10 μM AST. The cells were then left unstimulated or stimulated with insulin (100 nM) for 10 min. Cell lysates were immunoprecipitated (**A**,**B**) with an anti-p85 antibody and probed with anti-p85 and anti-insulin receptor substrate 1 (IRS-1) antibodies or analyzed for IR^Y1146^ phosphorylation (**C**,**D**). The density of the phosphorylated protein bands was analyzed by using NIH Image-J software and normalized by the arbitrary units of total proteins, ** *p* < 0.01. Data are expressed as the mean ± SD of three independent experiments.

S6K1 deficiency leads to IR sensitization in vitro and in vivo, as evidenced by increased AKT phosphorylation and IR tyrosine phosphorylation in the liver of S6K1 knockout mice [13,14]. Here, we tested if inhibition of S6K1 activity by AST could sensitize the IR and feedback activate the PI-3 kinase pathway. Co-immunoprecipitation with an antibody against the p85 subunit of PI-3 kinase revealed that insulin increased the binding of IRS-1 to the p85 subunit in 3T3-L1 adipocytes (Figure 4A) and L6 myotubes (Figure 4B). This binding was attenuated when the cells were incubated in the presence of high amino acid concentrations. AST treatment restored the binding of IRS-1 to the p85 subunit of PI-3 kinase in 3T3-L1 adipocytes and L6 myotubes cultured under high concentrations of amino acids to the levels comparable to that in the absence of amino acids (Figure 4A,B). The levels of total IRS-1 and p85 proteins in the cell lysates were equal, indicating that AST treatment does not alter the expression of IRS-1 and PI-3 kinase (Figure 4A,B). Insulin dramatically induced IR tyrosine phosphorylation, which was partially suppressed by incubation of the cells in the presence of high amino acid concentrations (Figure 4C,D). AST restored IR tyrosine phosphorylation in 3T3-L1 adipocytes and L6 myotubes to the levels equal to that in the absence of amino acids (Figure 4C,D).

### 2.7. AST Promotes GLUT4 Translocation to The Plasma Membrane

**Figure 5:**
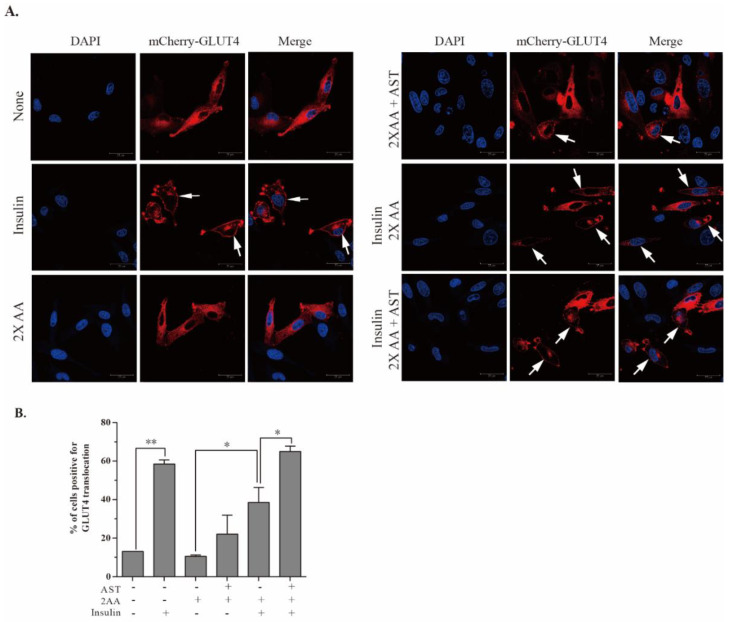
AST promotes GLUT4 translocation to the plasma membrane. (**A**) L6 cells transiently transfected with mCherry-GLUT4-myc were left untreated or treated with AST (10 μM) for 2 h in EBSS (containing 5% FBS) minus or plus 2× AA. The cells were then left unstimulated or stimulated with insulin (100 nM) for 10 min. The cells were fixed and stained with 4,6-diamidino-2-phenylindole (DAPI). mCherry-tagged GLUT4 red fluorescence was visualized under a Leica confocal microscope. GLUT4 translocation to the cytoplasm membrane was marked with arrows. (**B**) Quantification of GLUT4 translocation to the plasma membrane. The data represent the mean ± SD from one of three experiments with similar results. * *p* < 0.05; ** *p* < 0.01.

Insulin promotes glucose uptake by inducing the translocation of GLUT4 from the cytoplasm to the plasma membrane [32]. Here, we tested if AST could enhance GLUT4 translocation in L6 cells cultured under insulin resistance conditions. In unstimulated cells, red fluorescence protein (RFP)-tagged GLUT4 was evenly distributed in the cytoplasm. Upon insulin stimulation, the red fluorescence signals of GLUT4 were present on the periphery of the cells, e.g., the cell membrane. As shown in Figure 5A, insulin induced GLUT4 translocation to the plasma membrane in L6 cells cultured in the absence of amino acids. Insulin-induced GLUT4 translocation was suppressed when the cells were cultured in the presence of 2× AA. AST (10 μM) increased the percentage of membrane GLUT4-positive cells to the levels equivalent to that in the cells cultured without 2× AA. Enumeration of the GLUT4-positive cells revealed that AST significantly increased the percentage of L6 cells with GLUT4 expression on the cell membrane (Figure 5B).

### 2.8. AST Improves Glucose Uptake

**Figure 6:**
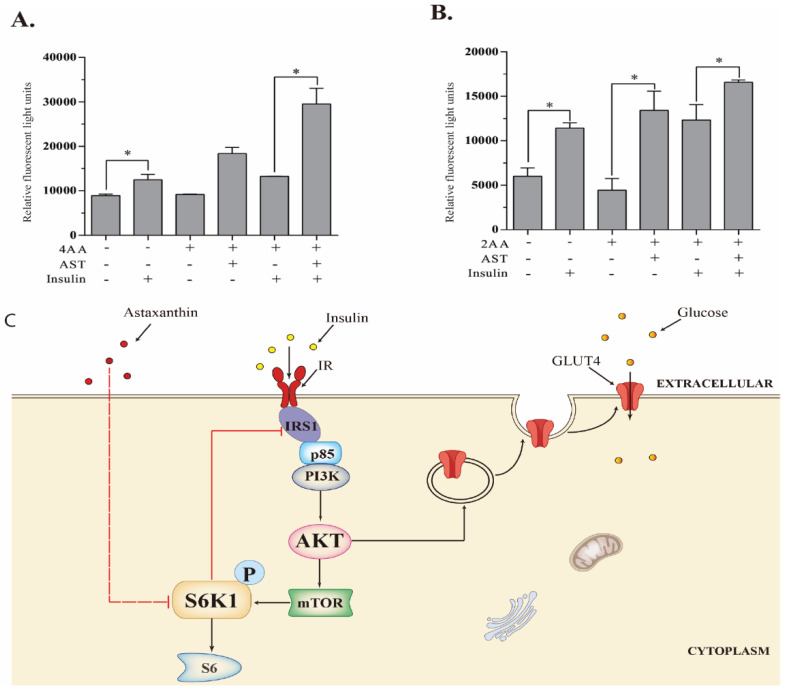
AST improves glucose uptake. 3T3-L1 adipocytes (**A**) and L6 myotubes (**B**) were starved of serum for 12 and 2 h, respectively. The cells were then incubated in the absence or presence of 4× or 2× AA or plus AST (10 μM) in EBSS for 1 h. The cells were pulsed with 2-NBDG (50 μM) in darkness for another 1 h and then left unstimulated or stimulated with insulin (100 nM) for 10 min. After aspirating the media, fluorescent signals were read in a microplate reader with excitation and emission wavelengths of 485 and 535 nm, respectively. Data represent the mean ± SD of one experiment in triplicate. * *p* < 0.05. (**C**) Schematic model depicting the mechanism of action of AST. S6K1 phosphorylates IRS-1^S1101^ and attenuates the activation of the PI-3 kinase pathway. AST inhibits S6K1, leading to decreased IRS-1^S1101^ phosphorylation and the sensitization of the PI-3 kinase pathway. AKT activation enhances GLUT4 translocation into the plasma membrane and improves glucose uptake.

Translocation of intracellular glucose transporters to the plasma membrane is essential for glucose uptake [33]. Finally, we tested if increased GLUT4 translocation by AST led to increased glucose uptake. As shown in Figure 6, insulin markedly increased the intracellular glucose levels in 3T3-L1 adipocytes and L6 myotubes by 39.9% and 89.9%, respectively. Insulin-induced glucose uptake was not significantly decreased when 3T3-L1 adipocytes were cultured in the presence of high concentrations of AA. AST increased the intracellular glucose levels in the insulin-stimulated 3T3-L1 adipocytes in the presence of high concentrations of amino acids (Figure 6A). Similar observations were made with L6 myotubes (Figure 6B).

## 3. Discussion

AST is a well-known nutritional supplement that helps to control blood glucose in individuals with diabetes mellitus [20,34,35]. AST offers a broad spectrum of other benefits to diabetic patients, including the suppression of inflammation and oxidative stress as well as the control of nonalcoholic fatty liver and diabetic complications [22,26,31,36]. AST functions as an antagonist of peroxisome proliferator-activated receptor (PPARγ) to exert its anti-inflammatory activity [37]. Our present study, focusing on the mechanisms of action of AST-mediated anti-hyperglycemic activity, has led to the identification of S6K1 as its molecular target. Our study provides mechanistic insights into how this nutritional supplement sensitizes the IR and improves glucose metabolism. 

S6K1, a well-recognized signaling molecule in the PI-3 kinase pathway, plays an important role in the pathogenesis of type 2 diabetes [38]. S6K1 is hyperactivated in the metabolic tissues such as liver, muscle, and fat under overnutrition conditions. Constitutively activated S6K1 desensitizes the IR by phosphorylating IRS-1^S1101^ and attenuating AKT activation [9,12]. S6K1^-/-^ mice fed a high-fat diet do not develop obesity and hyperglycemia increased AKT^T308^, AKT^S473^, and S6K1^T389^ phosphorylation [14]. S6K1 has been increasingly recognized as a potential therapeutic target for controlling insulin resistance-induced hyperglycemia [39,40]. PF-4708671, a specific S6K1 inhibiter, reduces glucose production in hepatocytes and increases glucose uptake in myocytes [40]. Our present study provides several lines of evidence that AST targets S6K1 to exert its anti-hyperglycemic effects: (1) AST inhibited the phosphorylation of two substrates of S6K1, S6^S235/236^, and IRS-1^S1101^; (2) AST induced feedback activation of the PI-3 kinase pathway, as evidenced by increased AKT^T308^, AKT^S473^, and S6K1^T389^ phosphorylation in 3T3-L1 adipocytes and L6 myotubes under insulin-sensitive and insulin-resistant conditions; (3) AST increased IR tyrosine phosphorylation and IRS-1 binding to the p85 subunit of the PI-3 kinase; (4) AST directly inhibited S6K1 activity in an in vitro kinase assay; (5) AST induced GLUT4 translocation to the plasma membrane and promoted glucose uptake in L6 cells; (6) AST was docked into the ATP-binding pocket of the enzymatic active center of S6K1. 

While these observations collectively suggest that AST is an inhibitor of S6K1, we noticed that the ability of AST to inhibit S6K1 activity in an in vitro kinase assay was slightly lower than its ability to inhibit S6K1 activity in cell culture. It is not clear whether AST could be enriched in the cytosol, thus improving its potency to inhibit S6K1 activity. Alternatively, AST may also target other signaling molecules to activate AKT. It should be noted that the plasma concentrations of AST in volunteers taking a single dose of AST reached approximately 2.1 μM at 6.7 h after oral administration [41]. Whether the concentrations of AST in the metabolic tissues in vivo are high enough to inhibit S6K1 activity remains uncertain; whether AST exerts its anti-hyperglycemic effects by targeting S6K1 needs to be further investigated. 

IRS-1 is a crucial adaptor protein that is phosphorylated at multiple serine and threonine residues by several kinases involved in insulin resistance [9,12]. IRS-1^S1101^ phosphorylation by S6K1 leads to its weak binding to the p85 subunit of PI-3 kinase and poor activation [16,42]. In addition, IRS-1^S1101^ phosphorylation also accelerates its degradation [43]. Chen et al. reported that A77 1726 and gingerenone A, two S6K1 inhibitors, inhibit IRS-1^S1101^ phosphorylation, sensitize the IR, and increase glucose uptake [16,44]. Ishiki et al. [30] reported that AST treatment of L6 myotubes leads to decreased phosphorylation of IRS-1 at serine 307, a site that is phosphorylated by c-Jun terminal kinase (JNK) [45]. Our study demonstrates that AST functioned as an S6K1 inhibitor to suppress IRS-1^S1101^ phosphorylation in 3T3-L1 adipocytes and in L6 myotubes under the insulin-sensitive and -resistant conditions. We provide further evidence that decreased IRS-1^S1101^ phosphorylation in AST-treated cells led to increased IRS-1 binding to the p85 subunit of PI-3 kinase (Figure 4A,B) and sensitized the IR, as evidenced by increased IR tyrosine phosphorylation (Figure 4 C,D). 

The PI-3 kinase pathway plays a central role in glucose metabolism [35]. Activation of AKT in this pathway triggers GLUT4 translocation in the plasma membrane, leading to increased glucose uptake [46,47,48,49]. AKT is phosphorylated by the protein kinase D (PKD) at S473 and by the mTOR complex 2 (mTORC2) at T308 [14]. IR signaling in insulin-resistant conditions such as high amino acid concentrations is desensitized, leading to poor PI-3 kinase and AKT activation [9,10]. It is well established that inhibition of S6K1 activity leads to feedback activation of the PI-3 kinase pathway and improved insulin signaling [9,12]. We recently reported that inhibition of S6K1 activity by A77 1726 and gingerenone A leads to increased AKT phosphorylation in 3T3-L1 adipocytes and L6 myotubes under normal and insulin-resistant conditions [16,44]. Several prior studies have shown that AST enhances AKT phosphorylation in L6 myotubes [30] and SH-SY5Y cells, a neuroblastoma cell line [31,50]. Our present study shows that AST inhibited S6K1 activity but increased AKT^T308^ and AKT^S473^ phosphorylation (Figure 1 and Figure 3). Moreover, we provide evidence that following AKT activation, GLUT4 was translocated into the cell membrane of L6 cells, and glucose uptake was increased in AST-treated cells. These findings support the notion that AST activates AKT by suppressing S6K1 activity and inducing feedback activation of the PI-3 kinase pathway. In contrast, other studies have shown that AST can inhibit AKT activation. For example, AST inactivates AKT, reduces hepatic lipid accumulation in high-fat-fed mice, enhances mitomycin C-induced cytotoxicity in human non-small cell lung cancer cells, and induces intrinsic apoptosis in oral cancer [36,51,52]. It is not clear if these seemingly contradicting observations are due to the lack of IR or insulin-like growth factor-1 (IGF-1) receptor expression in different cell types used in these studies. Alternatively, AST may target other signaling molecules that may lead to the inhibition of AKT phosphorylation. 

S6K1 inhibitors have been increasingly recognized as potential anti-diabetic drugs for treating T2DM. Several S6K1 inhibitors, such as PF-4708671, leflunomide, and gingerenone A, control hyperglycemia by sensitizing the IR and enhancing glucose uptake [16,40,44]. Leflunomide, a clinically approved anti-rheumatoid arthritis (RA) drug, has exhibited anti-hyperglycemic effects in RA patients [53,54]. Although numerous natural diets as well as their extracts have been used as anti-diabetic supplements, how they exert their anti-glycemic effects is poorly understood. Our study validates S6K1 as a new molecular target of AST and provides evidence that AST sensitizes insulin signaling to enhance GLUT4 translocation and glucose uptake. Our study sheds light on the mechanisms of AST-mediated IR sensitization. 

## 4. Materials and Methods

### 4.1. Reagents

AST, dexamethasone, 3-isobutyl-1-methylxanthine (IBMX), PF-4708671, 100-fold-concentrated amino acids stock (100× AA, 0.89 g/L l-alanine, 1.5 g/L l-asparagine · H_2_O, 1.33 g/L l-aspartic acid, 1.47 g/L l-glutamic acid, 0.75 g/L glycine, 1.15 g/L l-proline, 1.05 g/L l-serine), and 4,6-diamidino-2-phenylindole (DAPI) were purchased from Sigma Aldrich (St. Louis, MO, USA). Rosiglitazone was purchased from Calbiochem Research Biochemicals (Billerica, MA, USA). Insulin and 2-NBDG were purchased from Invitrogen Inc. (Grand Island, NY, USA). Dulbecco’s modified Eagle’s medium (DMEM), fetal bovine serum (FBS), and calf serum were purchased from HyClone Laboratories Inc. (Logan, UT, USA). ADP-Glo^TM^ kinase kit was purchased from Promega Corporation (Madison, WI, USA). Essential balanced salt solution (EBSS, 200 mg/mL CaCl_2_, 200 mg/mL MgSO_4_-7H_2_O, 400 mg/mL KCl, 2200 mg/mL NaHCO_3_, 6800 mg/mL NaCl, 140 mg/mL NaH_2_PO_4_-H_2_O) was purchased from Gibco (Life Technologies, Paisley, UK). TurboFect Reagent was purchased from Thermo Scientific (Waltham, MA, USA). Antibodies for phosphorylated proteins including AKT^S473^ (#4060), AKT^T308^ (#9275), S6K1^T389^ (#9234), S6^S235/236^ (#4858), IRS-1^S1101^ (#2385), and IR^Y1146^ (#3021) and their corresponding total proteins including AKT (#9272), S6K1 (#2708), S6 (#2217), IRS-1 (#2390), IR (#3025), and the p85 (#4257) subunit of the PI-3 kinase were purchased from Cell Signaling Technology (Danvers, MA, USA). The anti-β-actin monoclonal antibody was purchased from Santa Cruz Biotechnology. The GLUT4 expression vector (mCherry-Glut4-myc) was kindly provided by Dr. Amira Klip (The Hospital for Sick Children, Toronto, ON, Canada). 

### 4.2. Cell Lines and Differentiation

L6 and 3T3-L1 cells were purchased from the American Type Culture Collection (ATCC, Manassas, VA, USA) and cultured in complete DMEM with 10% FBS. For L6 myotube differentiation, L6 fibroblasts were cultured in DMEM containing 2% newborn bovine serum for 2 weeks, during which the same fresh medium was replenished every two days [16]. 3T3-L1 fibroblasts were differentiated into adipocytes according to Zebisch et al. [55]. Briefly, 3T3-L1 fibroblasts were seeded in 12-well plates. After incubation for 2–3 days, the confluent monolayers of 3T3-L1 cells were cultured in DMEM containing 500 μM IBMX, 0.25 μM dexamethasone, 2 μM rosiglitazone, 1 μg/mL insulin for 3 days. The media were then replenished with DMEM containing 1 μg/mL insulin every other day for 3 rounds. Approximately 95% of the cells were differentiated into adipocytes, which could be easily identified with the appearance of the “foam” cells under a light microscope and were confirmed by oil red staining. To model the insulin resistance condition, 3T3-L1 adipocytes and L6 myotubes were starved in serum-free DMEM overnight and 2 h respectively. 3T3-L1 adipocytes and L6 myotubes were then incubated for 4 h in EBSS containing 4-fold (4×) and 2-fold-concentrated (2×) amino acids (AA), respectively, in the absence or presence of AST (10 μM). The AA solution, which was manufactured as a 100-fold-concentrated stock, was diluted 1:25 and 1:50 for the final concentrations of 4× and 2× AA, respectively. 

### 4.3. Western Blot

Differentiated 3T3-L1 adipocytes and L6 myotubes were starved of serum overnight or for 2 h, respectively. The cells were treated with AST (2.5, 5, 10 μM) or PF (10 μM) for 4 h, followed by incubation with insulin (100 nM) for the indicated lengths of time. Untreated cells were included as a negative control (Figure 1). Cells were lysed in NP-40 lysis buffer (1% NP-40; 150 mM NaCl; 50 mM Tris-HCl, pH 8.0; 10 µg/mL aprotinin; 10 µg/mL leupeptin; 1 mM phenylmethylsulfonyl fluoride (PMSF); 5 mM ethylenediaminetetraacetic acid (EDTA); and 2 mM sodium vanadate). Equal amounts of proteins were separated by polyacrylamide gel electrophoresis and transferred onto a polyvinylidene difluoride membrane. The density of the bands was quantified using NIH Image-J software and normalized with the arbitrary units of their corresponding total proteins. The results represent the mean ± standard deviation (SD) of three independent experiments in bar graphs. To analyze the effect of AST on insulin signaling in 3T3-L1 adipocytes and L6 myotubes under insulin resistance conditions, these cells were pre-treated with high concentrations of amino acids as described above. The cell lysates were prepared and analyzed for protein phosphorylation (Figure 3 and Figure 4). 

### 4.4. Homology Modeling

The structure of S6K1 was modeled with an online Swiss-Model Server (Version 2020, Swiss Institute of Bioinformatics, Basel, Switzerland) [56]. The amino acid sequence of S6K1 (IDQ8BSK8) was obtained from UniProt (www.uniprot.org). S6K1 (Protein Data Bank [PDB] ID: 4L44) from *Homo sapiens* was chosen as a template. The predicted model of S6K1 was further assessed by Structural Analysis and Verification Server (SAVES v5.0, University of California at Los Angeles, Log Angeles, CA, USA).

### 4.5. Docking Modeling

The 3D structure of AST was obtained from the DrugBank database (https://www.drugbank.ca/). The pretreatment of the 3D structures of S6K1 and AST was performed by MGLTools 1.5.6 (The Scripps Research Institute, San Diego, CA, USA). The active and binding sites of S6K1 were determined by the NCBI CD-search tool (National Center for Biotechnology Information, U.S. National Library of Medicine, Bethesda, MD, USA). Software PyMOL 1.2 (Schrödinger, Inc., New York, NY, USA) was used to determine the docking pocket. The molecular docking of AST and S6K1 was performed by the AutoDock Vina 1.1.2 (The Scripps Research Institute, San Diego, CA, USA) [57].

### 4.6. In Vitro S6K1 Assay

An ADP-Glo^TM^ kinase kit was used to determine the ability of AST to inhibit S6K1 activity. Briefly, recombinant human S6K1 (25 ng per well) was mixed with AST (0, 10, 20, 40 μM) or PF-4708671 (10 μM) on ice for 20 min. S6K1 substrate and ATP (25 μM, final concentration) were added and incubated at room temperature for 1 h. ADP-Glo reagent (5 μL per well) was then added and incubated for 40 min. After the addition of the kinase detection buffer (10 μL per well) and incubation for 30 min, the fluorescence signals were measured in a TECAN luminescence plate reader. 

### 4.7. Co-Immunoprecipitation

Differentiated 3T3-L1 adipocytes and L6 myotubes were first starved in serum-free DMEM overnight or for 2 h, respectively. The cells were then incubated for 4 h in EBSS or EBSS containing 4× or 2× AA, respectively, in the absence or presence of 10 μM AST. The cells were left unstimulated or stimulated with insulin (100 nM) for 10 min. The cells were lysed in NP-40 lysis buffer and immunoprecipitated with an anti-p85 rabbit monoclonal antibody, followed by Western blot to detect p85 and IRS-1.

### 4.8. GLUT4 Translocation

L6 cells were transiently transfected with an expression vector encoding mCherry-Glut4-myc by using the TurboFect transfection reagent according to the manufacturer’s instruction. The cells were then incubated for 24 h. Approximately 70% of the cells displayed red fluorescence under a fluorescent microscope. The cells were left untreated or treated with AST (10 μM) for 4 h in EBSS containing 10% FBS with or without 2× AA. Cells were then left unstimulated or stimulated with 100 nM insulin for 30 min. The coverslips were fixed in 4% paraformaldehyde at room temperature for 10 min and then mounted with 50% glycerin in PBS containing 4,6-diamidino-2-phenylindole (DAPI) (0.5 μg/mL). mCherry-tagged Glut4 fluorescence was visualized under a Leica LP8 confocal microscope. The number of cells with GLUT4 membrane staining in 10 randomly selected fields from each treatment was divided by the total number of mCherry-GLUT4-positive cells. The percentage of the GLUT4 membrane-positive cells was calculated and presented in a bar graph. The results represent the mean ± SD of three independent experiments. 

### 4.9. Glucose Uptake

Intracellular glucose levels in 3T3-L1 adipocytes and L6 myotubes were measured using the fluorescent dye 2-NBDG. Differentiated 3T3-L1 and L6 cells seeded in 96-well plates were starved in serum-free DMEM for 12 and 2 h, respectively. 3T3-L1 and L6 cells were then cultured for 1 h in EBSS containing 4× or 2× AA, respectively, in the absence or presence of AST (10 μM). 2-NBDG (50 μM) was added and incubated in darkness for another 1 h. The cells were left unstimulated or stimulated with insulin 100 nM for 10 min. The cells were rinsed twice with PBS and then read for fluorescence intensity in a fluorescence microplate reader with excitation and emission wavelengths of 485 and 535 nm, respectively. The experiments were repeated twice with similar results. The data in Figure 6A,B represent the mean ± SD of one experiment in triplicate.

### 4.10. Statistical Analysis

One-way analysis of variance (ANOVA) (SPSS 22.0, SPSS Inc., Chicago, IL, USA). was used to determine the statistical significance of the differences in the density of Western blot bands (Figure 1) and in S6K1 activity (Figure 2) between the untreated control and the samples treated with the serially diluted AST (Figure 1). The unpaired Student’s *t* test was used to determine the statistical significance in the differences in the density of Western blot bands (Figure 3 and Figure 4), the percentage of the cells with GLUT4 expression on the cytoplasm membrane (Figure 5B), and glucose uptake (Figure 6A,B) between two groups. All the statistical analyses were performed on SPSS 22.0 (SPSS Inc., Chicago, IL, USA). A *p* value < 0.05 was considered statistically significant.
